# Tobacco, alcohol use and risk of hepatocellular carcinoma and intrahepatic cholangiocarcinoma: The Liver Cancer Pooling Project

**DOI:** 10.1038/s41416-018-0007-z

**Published:** 2018-03-09

**Authors:** Jessica L. Petrick, Peter T. Campbell, Jill Koshiol, Jake E. Thistle, Gabriella Andreotti, Laura E. Beane-Freeman, Julie E. Buring, Andrew T. Chan, Dawn Q. Chong, Michele M. Doody, Susan M. Gapstur, John Michael Gaziano, Edward Giovannucci, Barry I. Graubard, I-Min Lee, Linda M. Liao, Martha S. Linet, Julie R. Palmer, Jenny N. Poynter, Mark P. Purdue, Kim Robien, Lynn Rosenberg, Catherine Schairer, Howard D. Sesso, Rashmi Sinha, Meir J. Stampfer, Marcia Stefanick, Jean Wactawski-Wende, Xuehong Zhang, Anne Zeleniuch-Jacquotte, Neal D. Freedman, Katherine A. McGlynn

**Affiliations:** 1 Division of Cancer Epidemiology and Genetics, National Cancer Institute, Bethesda, MD USA; 2 Epidemiology Research Program, American Cancer Society, Atlanta, GA USA; 3 Division of Preventive Medicine, Department of Medicine, Brigham and Women’s Hospital, Boston, MA USA; 4 Channing Division of Network Medicine, Department of Medicine, Brigham and Women’s Hospital, Boston, MA USA; 5 Division of Gastroenterology, Massachusetts General Hospital and Harvard Medical School, Boston, MA USA; 6 Clinical and Translational Epidemiology Unit, Massachusetts General Hospital and Harvard Medical School, Boston, MA USA; 7 Division of Medical Oncology, National Cancer Centre Singapore, Singapore, Singapore; 8 VA Boston Healthcare System, Boston, MA USA; 9 Department of Epidemiology, Harvard T.H. Chan School of Public Health, Boston, MA USA; 10 Slone Epidemiology Center at Boston University, Boston, MA USA; 11 Department of Pediatrics, University of Minnesota, Minneapolis, MN USA; 12 Exercise and Nutrition Sciences, Milken Institute School of Public Health, George Washington University, Washington, DC USA; 13 Department of Nutrition, Harvard T.H. Chan School of Public Health, Boston, MA USA; 14 Department of Medicine and Obstetrics & Gynecology, Stanford University School of Medicine, Stanford, CA USA; 15 Department of Epidemiology and Environmental Health, University at Buffalo, Buffalo, NY USA; 16 Department of Population Health, New York University School of Medicine, New York, NY USA

**Keywords:** Risk factors, Cancer epidemiology

## Abstract

**Background:**

While tobacco and alcohol are established risk factors for hepatocellular carcinoma (HCC), the most common type of primary liver cancer, it is unknown whether they also increase the risk of intrahepatic cholangiocarcinoma (ICC). Thus, we examined the association between tobacco and alcohol use by primary liver cancer type.

**Methods:**

The Liver Cancer Pooling Project is a consortium of 14 US-based prospective cohort studies that includes data from 1,518,741 individuals (HCC *n* = 1423, ICC *n* = 410). Multivariable-adjusted hazards ratios (HRs) and 95% confidence intervals (CI) were estimated using proportional hazards regression.

**Results:**

Current smokers at baseline had an increased risk of HCC (hazard ratio (HR) = 1.86, 95% confidence interval (CI): 1.57–2.20) and ICC (HR = 1.47, 95% CI: 1.07–2.02). Among individuals who quit smoking >30 years ago, HCC risk was almost equivalent to never smokers (HR = 1.09, 95% CI: 0.74–1.61). Compared to non-drinkers, heavy alcohol consumption was associated with an 87% increased HCC risk (HR_≥7 drinks/day_ = 1.87, 95% CI: 1.41–2.47) and a 68% increased ICC risk (HR_≥5 drinks/day_ = 1.68, 95% CI: 0.99–2.86). However, light-to-moderate alcohol consumption of <3 drinks/day appeared to be inversely associated with HCC risk (HR_>0–<0.5 drinks/day_ = 0.77, 95% CI: 0.67–0.89; HR_>0.5–<1 drinks/day_ = 0.57, 95% CI: 0.44–0.73; HR_1–<3 drinks/day_ = 0.71, 95% CI: 0.58–0.87), but not ICC.

**Conclusions:**

These findings suggest that, in this relatively healthy population, smoking cessation and light-to-moderate drinking may reduce the risk of HCC.

## Introduction

The International Agency for Research on Cancer^1^ and the United States (US) Surgeon General^2^ have concluded that there is sufficient evidence to support a causal association between tobacco smoking and liver cancer. Similarly, alcohol is an established cause of liver cancer.^3,4^ However, few US studies have differentiated between the two dominant types of liver cancer: hepatocellular carcinoma (HCC, 75% of all liver cancers) and intrahepatic cholangiocarcinoma (ICC, 12%).^5–7^ Thus, it is unknown whether smoking and alcohol only increase the risk of HCC or whether they also increase the risk of ICC.

HCC usually develops in the background of oxidative stress and inflammation, triggered by chronic infection with hepatitis B or C virus (HBV or HCV), excess alcohol consumption, obesity, diabetes, and smoking.^8–10^ A recent meta-analysis suggested that cirrhosis, HBV/HCV, alcohol consumption, obesity, and diabetes are possible risk factors for ICC.^7^ However, results of studies examining the association between ICC and alcohol were heterogeneous, and the association between smoking and ICC remains unclear.^7^ In addition, the Surgeon General’s Report found heterogeneity of dose-response relationships between liver cancer and smoking intensity, pack-years, and duration.^2^

Evidence of an association between alcohol and liver cancer risk is based mainly on case–control studies, due to the rarity of liver cancer. However, 60–90% of HCCs develop in persons with pre-existing liver disease,^11,12^ which may lead persons to stop consuming alcohol and may bias the true association.^13^ Alternatively, case–control studies may overestimate the association due to recall bias among cases. Few prospective studies have been able to examine the association between alcohol consumption and HCC risk;^11^ only one such study has been conducted in the US.^14^ In addition, there has only been one case–control study of alcohol consumption and ICC in the US.^15^

To overcome the limitations of prior studies, we prospectively examined the association of smoking and alcohol use with HCC and ICC in a project that pooled data from 14 US-based cohort studies. In addition, we evaluated whether the associations varied by smoking duration, intensity, cessation, or alcohol type and amount.

## Patients and methods

### Study population

As described previously,^16^ all US-based cohort studies that are members of the National Cancer Institute (NCI) Cohort Consortium were invited to participate in the Liver Cancer Pooling Project (LCPP). All 14 participating studies contributed data on tobacco smoking and alcohol use: NIH-AARP Diet and Health Study (AARP),^17^ Agricultural Health Study (AHS),^18^ United States Radiologic Technologists (USRT) Study,^19^ The Breast Cancer Detection Demonstration Project (BCDDP),^20^ Prostate, Lung, Colorectal and Ovarian Cancer Screening Trial (PLCO),^21^ Women’s Health Study (WHS),^22^ Physicians’ Health Study (PHS),^23^ Health Professionals Follow-Up Study (HPFS),^24^ New York University Women’s Health Study (NYU),^25^ Cancer Prevention Study-II Nutrition Cohort (CPS-II),^26^ Iowa Women’s Health Study (IWHS),^27^ Black Women’s Health Study (BWHS),^28^ Women’s Health Initiative (WHI),^29^ and Nurses’ Health Study (NHS)^30^ (Supplementary Table 1). The individual cohorts were approved by the institutional review boards of the participating institutions; LCPP was approved by the NIH Office of Human Subjects Research. All participants provided informed consent prior to participation.

### Outcomes

Incident primary liver cancer (defined as International Classification of Diseases, 10th edition (ICD-10) diagnostic code C22) was ascertained by linkage to state cancer registries or medical/pathology record review. Cases were classified as HCC (International Classification of Diseases for Oncology, third edition (ICD-O-3) histology codes of 8170-8175), ICC (ICD-O-3 histology codes of 8032-8033, 8041, 8050, 8070-8071, 8140-8141, 8160, 8260, 8480, 8481, 8490, and 8560). Liver cancers other than HCC or ICC were excluded from the analysis (*n* = 313) as were cases missing histology information (*n* = 601). The current study included 1423 HCC cases, 410 ICC cases, and 1,516,908 non-cases.

### Exposure

Most studies defined smokers as persons who had ever smoked ≥100 cigarettes prior to study entry (Supplementary Table 2). However, a smoker was defined by USRT and BWHS as someone who regularly smoked (or smoked at least one cigarette per day) for 1 year, PLCO as someone who regularly smoked for 6 months, PHS and NHS as someone who ever smoked regularly, and NYU as someone who ever smoked. All studies assessed never/former/current smoking status and smoking intensity (cigarettes/day). Years since smoking cessation was not assessed by AHS, and duration of smoking was not assessed by AARP, PHS, and HPFS. In addition, pack-years of smoking were not able to be calculated for AARP and PHS. Smoking cessation, intensity, duration, and pack-years were examined as continuous variables for trends and categorised according to quartiles.

With the exceptions of BCDDP, NYU, and WHI, all studies assessed alcohol consumption over the past year (or 12 months) prior to study entry (Supplementary Table 3). BCDDP participants reported whether they ever consumed alcohol, and consumption frequency between ages 30 and 50. NYU conducted a retrospective assessment of “current” baseline alcohol consumption ~10 years after baseline. WHI assessed alcohol consumption over the past 3 months. To examine trends, the number of drinks for beer, wine, and liquor was provided by the parent-cohort, assumed to be a 12-ounce beer, 4-ounce wine, and 1-ounce liquor. Total alcohol consumption was categorised as >0–<0.5, 0.5–<1, 1–<3, 3–<5, 5–<7, ≥7 drinks/day for HCC, with ≥5 drinks/day as the highest category for ICC due to limited sample size. Non-drinkers were defined as those individuals reporting no alcohol consumption.

### Statistical analysis

Data were harmonised, as described above, and pooled for analysis. Cox proportional hazard regression analysis calculated adjusted hazards ratios (HRs) and 95% confidence intervals (CIs) for the associations of cigarette smoking and alcohol consumption with HCC and ICC, with follow-up time as the underlying time metric. Follow-up of the analytic cohort occurred from time at baseline until an event (i.e., incident liver cancer) or right-censoring (i.e., death, loss to follow-up, or last date of follow-up), whichever occurred first.

The proportional hazards assumption was tested using an interaction between smoking or alcohol use with log(time), as a continuous variable, in models that included confounders; no interactions were observed (*p* ≥ 0.05).

In addition to interaction between smoking and alcohol, effect measure modification by sex, body mass index (BMI, kg/m^2^), diabetes, and study was assessed. Departures from the null were assessed using likelihood ratio tests to compare regression models with and without a multiplicative term.^31^ Additive interaction was also assessed, but results were similar (data not shown).

On the basis of existing literature, potential confounders^31^ included alcohol consumption, smoking, age at questionnaire administration, race, sex, education, BMI, and diabetes. Variables remained in the adjusted model if they were associated with the exposure and outcome;^32^ all potential confounders met this criterion and were included in all final models, as was parent-cohort.

Tests of linear trend were conducted using continuous variables, excluding the referent group. To further describe the relationships of interest, we utilised cubic splines.^33^ For smoking-intensity splines, the reference was never smokers, with knots corresponding to quartiles of smoking intensity (0.5, 10, 15, and 25). For drinks-per-day splines, the reference was 0.25 drinks/day, with knots at the *a priori* categories of 1, 3, 5, and 7 drinks/day.

All analyses were conducted using SAS version 9.3 (SAS Institute, Cary, NC, USA). All *p*-values are two-sided.

### Nested case–control study of HBV/HCV

Serum samples for determination of HBV and HCV status were available from a subset of participants. To determine HBV status, hepatitis B surface antigen (HBsAg) was assayed using the Bio-Rad GS HBsAg 3.0 enzyme immunoassay (Bio-Rad Laboratories, Redmond, WA, USA). To determine HCV status, antibody to hepatitis C virus (anti-HCV) was assessed using the Ortho HCV Version 3.0 ELISA test system (Ortho-Clinical Diagnostics, Inc.).

### Sensitivity analyses

Lag analyses, excluding the first 5 years of follow-up, were conducted, and we also stratified by quartiles of follow-up time. Additionally, we analysed together confirmed or suspected HCC cases, which included histologically classified HCC cases and additional suspected HCC cases defined as ICD-O-3 histology codes of 8000, 8010, or missing. Finally, we excluded non-drinkers from our analyses and utilised >0–<0.5 as the referent group.

## Results

Demographic characteristics of cases and non-cases are shown in Table 1. Compared with non-cases, individuals who developed HCC or ICC were more likely to be older, male, overweight or obese, and have diabetes.

**Table 1:** Characteristics of participants in the Liver Cancer Pooling Project

	Non-cases (*N* = 1,516,908)	HCC (*N* = 1423)	ICC (*N* = 410)	
	*N* (%)	*N* (%)	*N* (%)	
*Age at entry*				
<50	219,059 (14.4)	33 (2.3)	10 (2.4)	
50–59	502,903 (33.2)	364 (25.6)	102 (24.9)	
60–69	667,261 (44.0)	899 (63.2)	249 (60.7)	
≥70	127,649 (8.4)	127 (8.9)	49 (12.0)	
Missing	36	0	0	
*Sex*				
Male	603,416 (39.8)	1,039 (73.0)	223 (54.4)	
Female	913,492 (60.2)	384 (27.0)	187 (45.6)	
*Race*				
White	1,328,057 (88.6)	1,175 (84.6)	364 (89.7)	
Black	112,666 (7.5)	75 (5.4)	14 (3.4)	
Asian/Pacific Islander	18,846 (1.3)	33 (2.4)	7 (1.7)	
American Indian/Alaskan Native	3,319 (0.2)	5 (0.4)	3 (0.7)	
Other	36,333 (2.4)	101 (7.3)	18 (4.4)	
Missing	17,687	34	4	
*Body mass index* *(kg/m* ^*2*^ *)*				
<18.5	16,033 (1.1)	11 (0.8)	1 (0.3)	
18.5–24.9	594,061 (40.2)	346 (25.0)	122 (30.7)	
25–29.9	567,257 (38.4)	578 (41.8)	169 (42.6)	
≥30	300,013 (20.3)	447 (32.3)	105 (26.4)	
Missing	39,544	41	13	
*Education*				
Less than High School	82,207 (5.6)	124 (9.1)	33 (8.2)	
High School degree	275,237 (18.9)	283 (20.7)	69 (17.2)	
Some College/vocational	451,277 (31.0)	423 (31.0)	125 (31.1)	
College degree	314,268 (21.6)	259 (19.0)	101 (25.1)	
Graduate degree	332,474 (22.8)	276 (20.2)	74 (18.4)	
Missing	61,450	58	8	
*Diabetes*				
No	1,401,955 (93.3)	1060 (74.9)	358 (88.0)	
Yes	100,819 (6.7)	356 (25.1)	49 (12.0)	
Missing	14,134	7	3	
*Cohort*				
NIH-AARP Diet and Health Study	564,499 (37.2)	910 (64.0)	235 (57.3)	
Agricultural Health Study	34,729 (2.3)	10 (0.7)	2 (0.5)	
US Radiologic Technologists Study	72,402 (4.8)	4 (0.3)	1 (0.2)	
Breast Cancer Demonstration Project	51,624 (3.4)	8 (0.6)	6 (1.5)	
Prostate, Lung, Colorectal, and Ovarian Cancer Screening Trial	149,635 (9.9)	154 (10.8)	42 (10.2)	
Women’s Health Study	39,840 (2.6)	6 (0.4)	5 (1.2)	
Physicians’ Health Study	28,978 (1.9)	28 (2.0)	0 (0.0)	
Health Professionals Follow-Up	51,388 (3.4)	32 (2.3)	14 (3.4)	
NYU Women’s Health Study	14,250 (0.9)	5 (0.4)	3 (0.7)	
Cancer Prevention Study-II	160,394 (10.6)	115 (8.1)	31 (7.6)	
Iowa Women’s Health Study	28,570 (1.9)	29 (2.0)	10 (2.4)	
Black Women’s Health Study	57,152 (3.8)	6 (0.4)	1 (0.2)	
Women’s Health Initiative	160,988 (10.6)	67 (4.7)	46 (11.2)	
Nurses’ Health Study	102,459 (6.8)	49 (3.4)	14 (3.4)	

**Fig. 1:**
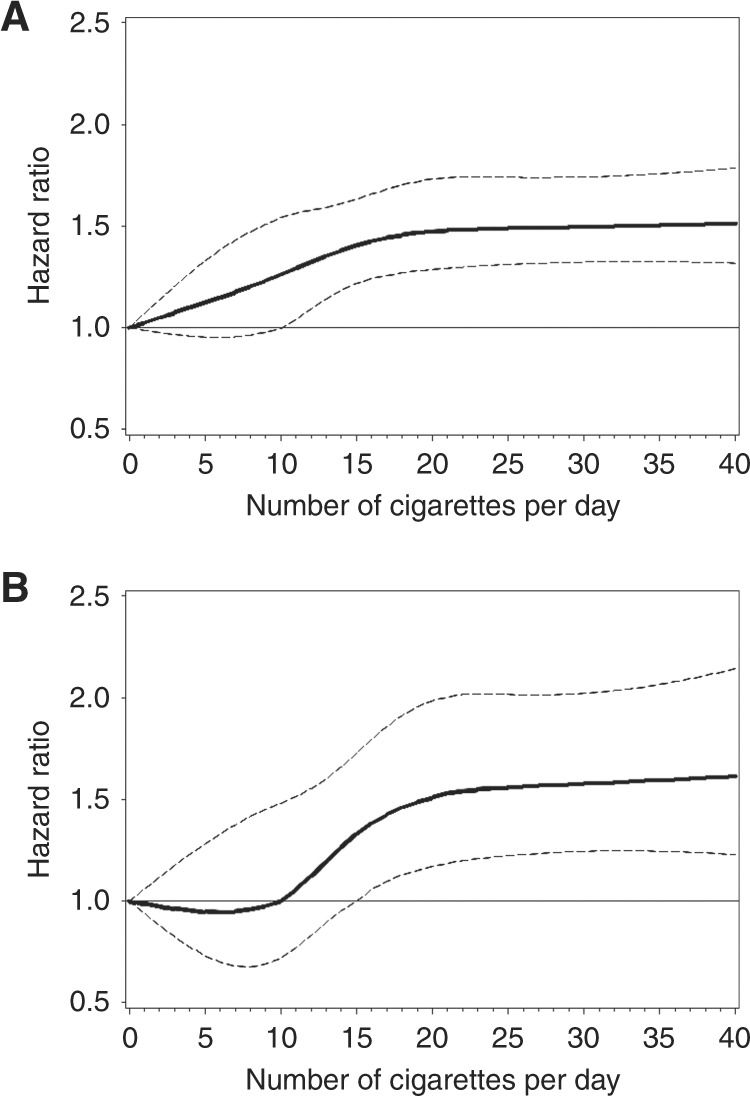
Cubic spline graph of the multivariate-adjusted HRs (represented by the solid line) and 95% CIs (represented by the dotted lines) for the association between smoking intensity and **a** HCC and **b** ICC in the Liver Cancer Pooling Project (knots: 0.5, 10, 15, 25; referent: 0)

Former and current smokers had increased risks of HCC and ICC, compared to never smokers (Table 2). Smoking more than 25 cigarettes per day was associated with a 55% increased HCC risk (95% CI: 1.30–1.84) and an 86% increased ICC risk (95% CI: 1.37–2.53). Restricted cubic regression splines show the increasing risk for both HCC and ICC associated with increasing levels of smoking intensity (Fig. 1a, b).

**Table 2:** Adjusted* hazards ratios and 95% confidence intervals for associations between cigarette smoking and hepatocellular carcinoma and intrahepatic cholangiocarcinoma incidence, Liver Cancer Pooling Project

Cigarette smoking		Hepatocellular carcinoma			Intrahepatic cholangiocarcinoma		
	Non-case, *N*	Case, *N*	HR	95% CI	Case, *N*	HR	95% CI
*Smoking status*							
Never smoker	591,378	360	1.00		124	1.00	
Former smoker	507,215	588	1.24	(1.08−1.43)	177	1.32	(1.03–1.68)
Current smoker	209,556	242	1.86	(1.57–2.20)	60	1.47	(1.07–2.02)
*Smoking cessation, years*							
Never smoker	573,113	358			122		
>2–10	112,879	123	1.47	(1.19–1.82)	43	1.68	(1.17–2.42)
>10–20	257,299	383	1.18	(1.00–1.38)	100	1.16	(0.86–1.55)
>20–30**	58,229	39	1.21	(0.85–1.71)	27	1.42	(0.90–2.25)
>30	46,028	31	1.09	(0.74–1.61)			
*p* for trend***				0.003			0.5
*Smoking intensity (No./day)*							
Never smoker	591,381	360	1.00		124	1.00	
≤10	218,375	170	1.16	(0.96–1.40)	50	1.00	(0.72–1.40)
>10–15	123,753	197	1.44	(1.19–1.73)	46	1.19	(0.83–1.71)
>15–25	173,570	196	1.46	(1.21–1.74)	55	1.39	(1.00–1.93)
>25	150,311	240	1.55	(1.30–1.84)	74	1.86	(1.37–2.53)
*p* for trend***				0.02			0.02
*Duration of smoking, years*							
Never smoker	373,171	133	1.00		54	1.00	
≤12	91,080	32	1.03	(0.70–1.53)	16	1.42	(0.80–2.51)
>12–22	84,642	50	1.46	(1.04–2.04)	14	1.24	(0.68–2.27)
>22–34	86,851	53	1.32	(0.95–1.84)	15	1.18	(0.66–2.12)
>34	85,951	67	1.50	(1.10–2.03)	30	2.01	(1.26–3.20)
*p* for trend***				0.03			0.2
*Pack-years*							
Never smoker	394,092	139	1.00		57	1.00	
≤7	99,635	35	1.06	(0.73–1.55)	17	1.25	(0.72–2.17)
>7–17	77,516	34	1.14	(0.78–1.67)	15	1.47	(0.82–2.62)
>17–34	88,416	75	1.90	(1.42–2.55)	20	1.57	(0.93–2.65)
>34	89,506	75	1.47	(1.08–1.98)	30	2.04	(1.28–3.27)
*p* for trend***				0.7			0.06

CI confidence interval, HR hazard ratio. *Adjusted for: sex, age (continuous), race (white, black, Asian/Pacific Islander, American Indian/Alaskan Native, other), cohort (AARP, AHS, USRT, BCDDP, PLCO, WHS, PHS, HPFS, CPS-II, NYU, IWHS, BWHS, WHI, NHS), BMI (continuous), diabetes (yes/no), alcohol (non-drinker, and >0–<0.5, 0.5–<1, 1–<3, 3–<5, 5–<7, ≥7 drinks/day), and education (less than HS, HS, some college/vocational, college degree, post-college)

**Highest category considered for ICC. This corresponds to >20 years since quitting smoking

***p-value for trend of continuous variable, excluding the referent group

Also shown in Table 2, increasing years since smoking cessation was associated inversely with HCC risk (*p*-trend = 0.003), with persons who ceased smoking >30 years ago having a similar HCC risk as never smokers. Smoking cessation was not associated with ICC in the same dose-dependent manner (*p*-trend = 0.5).

**Fig. 2:**
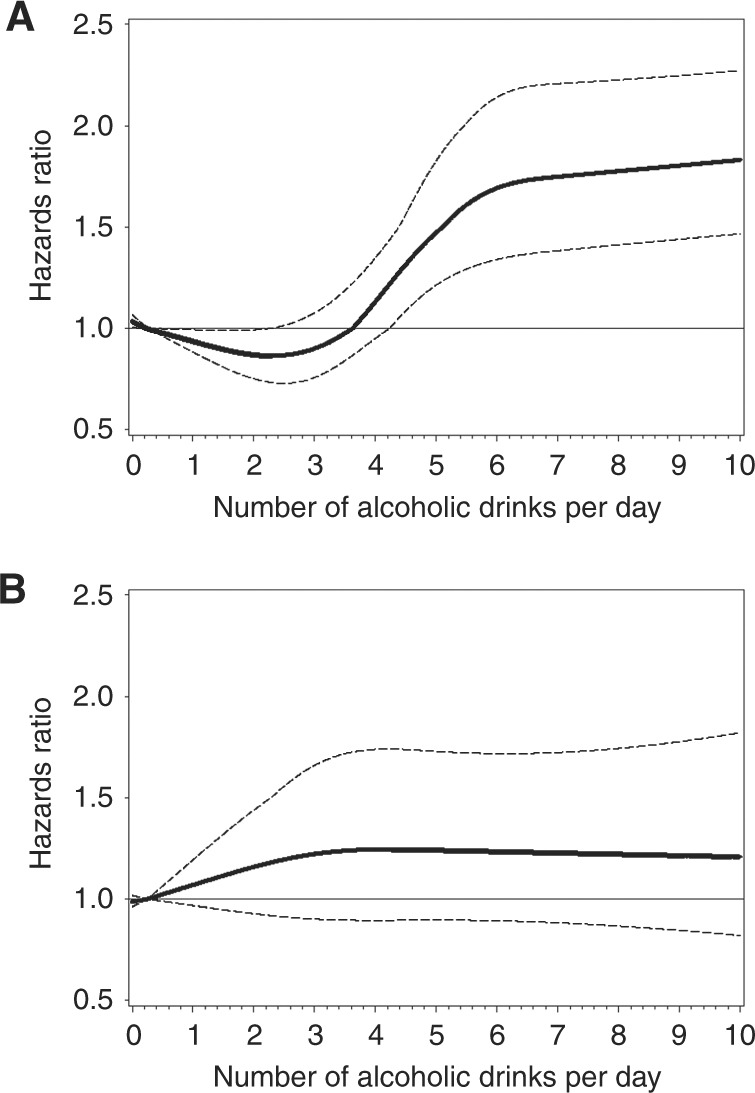
Cubic spline graph of the multivariate-adjusted HRs (represented by the solid line) and 95% CIs (represented by the dotted lines) for the association between drinks per day of alcohol and **a** HCC (knots: 1, 3, 5, and 7; referent: 0.25) and **b** ICC (knots: 1, 3, and 5; referent: 0.25) in the Liver Cancer Pooling Project

As shown in Table 3, the highest amounts of any alcohol consumption were associated with an 87% increased HCC risk (95% CI: 1.41–2.47), and a 68% increased ICC risk (95% CI: 0.99–2.86). Cubic splines show that HCC risk increased at ~4 drinks per day of alcohol, but ICC risk increased modestly with any amount of alcohol (Fig. 2a, b). High levels of beer, liquor, or wine consumption were associated with increased HCC and ICC risks, but estimates lacked precision. However, <3 drinks per day of alcohol was associated with a 23–43% decreased HCC risk (Table 3 and Fig. 2a). In the sensitivity analysis excluding non-drinkers, consumption of 0.5–<1 drink per day was associated with a 26% decreased HCC risk (95% CI: 0.58–0.95), compared to consumption of >0–<0.5 drinks per day (Supplementary Table 4).

**Table 3:** Adjusted* hazards ratios and 95% confidence intervals for associations between alcohol intake and hepatocellular carcinoma and intrahepatic cholangiocarcinoma incidence, Liver Cancer Pooling Project

Type of alcohol	Hepatocellular carcinoma				Intrahepatic cholangiocarcinoma			
	Non-case, *N*	Case, *N*	HR	95% CI	Non-case, *N*	Case, *N*	HR	95% CI
*Any alcohol*								
Non-drinker	3,34,649	342	1.00		334,649	79	1.00	
Drinks/day								
>0–<0.5	562,675	443	0.77	(0.67–0.89)	562,675	154	1.06	(0.80–1.40)
0.5–<1	126,280	73	0.57	(0.44–0.73)	126,280	33	1.02	(0.67–1.54)
1–<3	171,049	148	0.71	(0.58–0.87)	171,049	50	1.02	(0.70–1.48)
3–<5	35,851	67	1.04	(0.79–1.36)	35,851	15	1.24	(0.70–2.18)
5–<7**	13,200	28	1.00	(0.68–1.49)	26,886	18	1.68	(0.99–2.86)
≥7	13,686	62	1.87	(1.41–2.47)	—	—	—	
*p* for trend***				<0.0001				0.5
*Beer*								
Non-drinker	535,947	472	1.00		535,947	139	1.00	
Drinks/day								
>0–<0.5	435,229	446	0.84	(0.73–0.96)	435,229	133	0.99	(0.77–1.27)
0.5–<1	34,929	40	0.92	(0.66–1.28)	34,929	9	0.82	(0.41–1.64)
1–<3	38,887	53	0.86	(0.64–1.15)	38,887	11	0.81	(0.43–1.52)
3–<5**	12,172	31	0.97	(0.66–1.43)	20,801	10	1.27	(0.66–2.47)
≥5	8,629	36	1.55	(1.09–2.20)	—	—	—	
*p* for trend***				0.001				0.9
*Liquor*								
Non-drinker	524,853	536	1.00		524,853	134	1.00	
Drinks/day								
>0–<0.5	422,325	362	0.81	(0.70–0.93)	422,325	123	1.08	(0.84–1.39)
0.5–<1	35,099	27	0.69	(0.47–1.02)	35,099	13	1.10	(0.62–1.97)
1–<3	59,608	88	1.12	(0.89–1.41)	59,608	17	0.88	(0.52–1.47)
3–<5**	14,622	29	1.20	(0.82–1.75)	24,609	15	1.40	(0.81–2.41)
≥5	9,987	36	1.22	(0.86–1.73)				
*p* for trend***				0.0004				0.3
*Wine*								
Non-drinker	454,866	557	1.00		454,866	134	1.00	
Drinks/day								
>0–<0.5	478,686	408	0.74	(0.65–0.85)	478,686	120	0.81	(0.63–1.05)
0.5–<1	68,874	49	0.66	(0.49–0.89)	68,874	24	1.18	(0.75–1.84)
1–<2	42,471	33	0.67	(0.47–0.96)	42,471	13	0.93	(0.52–1.66)
≥2	18,453	30	1.35	(0.93–1.96)	18,453	11	1.82	(0.97–3.39)
*p* for trend***				<0.0001				0.03

CI confidence interval, HR hazard ratio. *Adjusted for: sex, age (continuous), race (white, black, Asian/Pacific Islander, American Indian/Alaskan Native, other), cohort (AARP, AHS, USRT, BCDDP, PLCO, WHS, PHS, HPFS, CPS-II, NYU, IWHS, BWHS, WHI, NHS), BMI (continuous), diabetes (yes/no), cigarette smoking (never, current, former), smoking intensity (cigarettes/day) education (less than HS, HS, some college/vocational, college degree, post-college)

**Highest category considered for ICC. This corresponds to ≥3 drinks/day for beer and liquor, and ≥5 drinks/day for any alcohol

***P-value for trend of continuous variable, excluding the referent group

There was no evidence of effect measure modification by sex, BMI, or smoking (*p* ≥ 0.05) on the association between alcohol and HCC. However, when we examined the interaction of alcohol and diabetes, there was some evidence of effect measure modification (*p* = 0.01) (Supplementary Table 5). Among the group of individuals that consumed >5 drinks per day, HCC risk was greater in those with diabetes (HR = 1.87, 95% CI: 1.21–2.89) than in those without diabetes (HR = 1.33, 95% CI: 1.00–1.78). There was no effect measure modification of the associations between smoking and HCC (Supplementary Table 6). We also examined parent-cohort as a possible effect measure modifier but found no evidence (*p*-interaction ≥0.05). There was no effect measure modification of the associations between smoking or alcohol and ICC (Supplementary Tables 7–8).

Results from a 5-year lag analysis were not substantially different than those from our main stratified model (Supplementary Tables 9–10). In addition, examination of quartiles of follow-up time did not result in notable differences (Supplementary Table 11). When we included suspected HCC cases, results did not differ from our main analysis of confirmed cases (Supplementary Tables 12–13).

Among the HCC cases tested (*n* = 188), 49 (26.1%) were positive for anti-HCV and 14 (7.5%) were positive for HBsAg. Among the matched controls (*n* = 462), 11 (2.4%) were positive for anti-HCV and 5 (1.1%) were positive for HBsAg. There was no association between HCV or HBV status and smoking or alcohol consumption (data not shown).

## Discussion

In the present study, current smoking was associated with 47–86% increased HCC and ICC risks, while increasing years since smoking cessation was associated inversely with HCC risk only. Heavy alcohol consumption was associated with 68–87% increased HCC and ICC risks. Light or moderate alcohol consumption (i.e., <3 drinks per day) was associated with a decreased HCC, but not ICC, risk. A positive multiplicative interaction was observed between heavy alcohol consumption and diabetes with HCC risk.

The current study is the largest US study of liver cancer to date with detailed information on smoking history. Previous attempts to determine the association between smoking and ICC in the US have been limited by small sample size^15^ or determination of smoking from ICD-9 codes,^34–36^ which results in low sensitivity and exposure misclassification.^37^ A recent meta-analysis of smoking and ICC estimated a 31% increased risk among individuals with a history of smoking, but there was high heterogeneity among studies.^7^ In the current study, smoking was consistently associated with increased ICC risk, regardless of the definition used. We also report that smoking is associated with increased risk of HCC. Smoking cessation was inversely associated with HCC risk. The lack of association with ICC could be due to limited statistical power, as the association between smokers that quit more than 10 years prior and ICC risk was not statistically different than that of never smokers.

A meta-analysis^5^ and the Surgeon General’s Report^2^ found a 51–70% increased liver cancer risk for current smoking. However, little to no association was noted between former smoking and liver cancer risk, and the associations were consistent when stratified by study design, geographic region, sample size, and publication period.^5^ In the current study, we report the largest increase in risk for current smokers, but also found increased risks of both HCC and ICC associated with former smoking. However, HCC risk returned to near non-smoking levels after ceasing smoking for >30 years.

Tobacco carcinogens are metabolised in the liver^38^ and experimental studies have identified several constituents of tobacco smoke as hepatocarcinogens (e.g., 2-acetylaminofluorene and 4-aminobiphenyl).^39^ Although it is unclear whether DNA adducts are sufficient to cause tumourigenesis, formation of DNA adducts in hepatocytes may be an important initiator of tumourigenesis.^39^ Studies have also shown levels of 4-aminobiphenyl-DNA adducts^40^ and polycyclic aromatic hydrocarbon-DNA adducts^41^ are increased in HCC tissue compared to normal liver tissue, suggesting that these tobacco carcinogens could play a role in HCC development.

Two recent meta-analyses have reported heavy alcohol consumption is associated with liver cancer risk,^11,42^ similar to our results. The first meta-analysis examined only prospective studies and reported that heavy drinking (≥3 drinks/day) was associated with a 16% increased risk. Moderate drinking (<3 drinks/day) was associated with a 34% reduced risk only among non-Asian populations.^11^ In Asian populations, the association with moderate drinking was null.^11^ This is perhaps due to suggested synergism between viral hepatitis and alcohol consumption,^43,44^ as HBV and HCV are more prevalent in some Asian countries. The second meta-analysis examined both prospective and retrospective studies and reported that heavy drinking (>4 drinks/day) was associated with a two-fold increased liver cancer risk, which was more pronounced for case–control than cohort studies. No association with liver cancer was noted for light (≤1 drink/day) or moderate drinking (≤4 drinks/day).^42^

In contrast to these meta-analyses, we report an inverse relationship between moderate drinking (i.e., up to 3 drinks/day) and liver cancer risk. There are two possible explanations for this. The non-drinker group could include former drinkers who developed liver disease. However, when we performed a lag analysis to account for possible pre-existing liver disease, excluding cases that developed within the first five years, our results were similar. Alternatively, moderate alcohol consumption is associated with a decreased risk of type II diabetes, possibly through increased insulin sensitivity.^45^ As diabetes is a risk factor for HCC,^8,55^ moderate alcohol consumption could decrease HCC risk via a decrease in the risk of diabetes. Among individuals without diabetes, moderate alcohol consumption was associated with a 35% decreased HCC risk, while among individuals with diabetes, there was a null association. Two other studies have examined the interactions between alcohol consumption and diabetes on HCC risk. Similar to our current report, these studies reported an interaction.^43,44^

One prior meta-analysis of ICC risk factors reported a 3-fold increased risk of ICC associated with heavy alcohol consumption, which was primarily defined as ~6 drinks/day or alcoholic liver disease.^7^ Similarly, we report that drinking ≥5 drinks/day is associated with a 68% increased ICC risk. However, unlike HCC, moderate alcohol consumption did not decrease ICC risk. Overall, there was no evidence of interaction between diabetes and alcohol for ICC (*p* = 0.3). However, among individuals without diabetes, there was no increased risk of ICC with moderate drinking. For individuals with diabetes, there was a suggested increased risk of ICC with moderate drinking. Thus, this study suggests that alcohol may have a different mechanism of action in the ICC carcinogenic process compared to HCC. In laboratory studies of tumour tissue, it has been shown that both HCC and ICC have high rates of glycolysis, but the mechanisms involved in glucose uptake and glycolytic metabolism differ by cell of origin.^46,47^

Alcohol may contribute to carcinogenesis through mechanisms of acetaldehyde, the first metabolite of ethanol oxidation, interfering with DNA synthesis and repair; induction of CYP2E1, which metabolises ethanol to acetaldehyde, increasing reactive oxygen-species production, lipid peroxidation, and DNA damage; alteration of the antioxidant defense systems and inhibition of DNA repair; disruption of the methyl group transfer; decreased hepatic retinoic acid; and iron overload leading to DNA strand breaks and p53 mutations.^48,56^ Diabetes is thought to influence the development of cancer through cytokines, such as tumour necrosis factor-alpha and interleukin-6, by decreasing apoptosis and causing uncontrolled proliferation of hepatocytes.^49^ Both alcohol consumption and diabetes may promote hepatocarcinogenesis through chronic inflammation resulting in increased oxidative stress.^48,49^ Thus, it has been postulated that alcohol-induced oxidative stress may increase susceptibility to development of diabetes, or increase the susceptibility of persons with diabetes to cirrhosis, or both.^43,50^ In our study, we report greater HCC risk for ≥5 drinks per day among individuals with diabetes.

Previous studies have reported synergistic effects of smoking and alcohol consumption,^51,52^ but we did not find evidence of an interaction between smoking and alcohol (*p* = 0.8). While the reason for differences between the current study and prior literature is unclear, we hypothesise that it may be due to differences in the study populations. Prevalence of HBV or HCV was 60–75% in the case groups of previously published studies.^51,52^ In the current study, only 32% of tested cases were HBV or HCV positive. Kuper et al.^51^ reported that the synergistic interaction was more evident among individuals without HBV or HCV, but the sample size was limited (*n* = 83).

While it has been proposed that case–control studies may underestimate the alcohol-liver cancer association due to pre-existing liver disease, as persons with liver disease may be advised to stop drinking,^11^ the positive association between alcohol consumption and liver cancer risk is higher in case–control than cohort studies.^42^ Thus, case–control studies may overestimate the association due to recall bias among cases. Alternatively, case–control studies may enroll heavier drinkers or quantify lifetime alcohol consumption better than cohort studies, as cohort studies generally ask about alcohol consumption at baseline or within the past 12 months. Cohort studies may also underestimate the association due to pre-existing liver disease.^11^ However, when we performed a lag analysis to account for possible pre-existing liver disease, excluding cases that developed within the first 5 years, our results were similar. We also performed an analysis stratified by follow-up time, and the results were similar across the quartiles of follow-up time. The association may also be underestimated in cohort studies if a sizable proportion of the population stops drinking during follow-up. However, it is estimated that drinking must have ceased for at least 20 years for liver cancer risk to be equivalent to the risk among never drinkers.^53^ In addition, individuals with liver disease who drink heavily may die from liver-related complications prior to potential development of liver cancer. While we did not run competing risk analyses, our primary aim was to estimate cause-specific relative risk, and death from liver disease was treated as a censored event. Estimation of cause-specific relative risk does not require independence of the outcome and competing events to obtain valid relative risk estimates.^54^

In the current study, data on exposures and some potential confounders were based on self-report and HBV/HCV status was not available for all individuals. However, for individuals with HBV or HCV status available, there was no association between these potential covariates and the exposures of smoking or alcohol consumption. This suggests that HBV and HCV are not confounders of the association between smoking or alcohol consumption and HCC or ICC. Information on use of pipes and cigars was also not available, and the cohorts included primarily white, older, non-Hispanic participants. However, this study had a sufficient sample size to evaluate the association between smoking and alcohol and incidence of HCC and ICC, and allow examination of interactions between categories of smoking or alcohol consumption and HCC risk factors.

In conclusion, our findings suggest that smoking and heavy alcohol consumption increase liver cancer risk. However, smoking cessation was associated with decreasing HCC risk in a dose-dependent manner, and light-to-moderate alcohol consumption was associated with decreased HCC risk.

## Electronic supplementary material

Supplemental Material
