# Supplementary material for: Tobacco, alcohol use and risk of hepatocellular carcinoma and intrahepatic cholangiocarcinoma: The Liver Cancer Pooling Project
Source: Br J Cancer. 2018 Mar 9;118(7):1005–12. doi: 10.1038/s41416-018-0007-z (PMC5931109; doi:10.1038/s41416-018-0007-z)
Supplement: Supplementary file 1 — Supplemental Material [file 41416_2018_7_MOESM1_ESM.doc]

**Supporting Table S1.** Cohorts Participating in the Liver Cancer Pooling Project.

| Cohort | Location | Recruitment/ Follow-up | Cohort  Characteristics | Non-Cases Total | |  | HCC Total | |  | ICC Total | |
| --- | --- | --- | --- | --- | --- | --- | --- | --- | --- | --- | --- |
|  |  |  |  | Men | Women |  | Men | Women |  | Men | Women |
| NIH-AARP Diet and  Health Study | multiple states | 1995/2008 | AARP members | 564,499 | |  | 910 | |  | 235 | |
|  |  |  |  | 338,234 | 226,265 |  | 752 | 158 |  | 161 | 74 |
| Agricultural Health  Study | North Carolina,  Iowa | 1999/2008 | Farmers and spouses | 34,729 | |  | 10 | |  | 2 | |
|  |  |  |  | 18,974 | 15,786 |  | 5 | 5 |  | 2 | 0 |
| United States  Radiologic Technologists  Study | multiple states | 1994/2008 | U.S. radiologic technologists | 72,402 | |  | 4 | |  | 1 | |
|  |  |  |  | 16,112 | 56,290 |  | 2 | 2 |  | 1 | 0 |
| The Breast Cancer  Demonstration Project | multiple states | 1987/1999 | Breast cancer screening program | 51,624 | |  | 8 | |  | 6 | |
|  |  |  |  | - | 51,624 |  | - | 8 |  | - | 6 |
| Prostate, Lung,  Colorectal, and  Ovarian Cancer  Screening Trial | multiple states | 1993/2009 | Trial of cancer screening modalities | 149,635 | |  | 154 | |  | 42 | |
|  |  |  |  | 73,607 | 76,028 |  | 131 | 23 |  | 26 | 16 |
| Women's Health Study | multiple states | 1993/2010 | Trial of low-dose aspirin and vitamin  E in prevention of CVD | 39,840 | |  | 6 | |  | 5 | |
|  |  |  |  | - | 39,840 |  | - | 6 |  | - | 5 |
| Physicians’ Health Study | multiple states | 1981/2010 | Physicians | 28,978 | |  | 28 | |  | 0 | |
|  |  |  |  | 28,978 | - |  | 28 | - |  | 0 | - |
| Health Professionals Follow-Up Study | multiple states | 1986/2010 | Male health professionals,  aged 40-75 | 51,388 | |  | 32 | |  | 14 | |
|  |  |  |  | 51,388 | - |  | 32 | - |  | 14 | - |
| NYU Women's Health  Study | New York | 1985/2010 | Study of hormones and breast  cancer | 14,250 | |  | 5 | |  | 3 | |
|  |  |  |  | - | 14,250 |  | - | 5 |  | - | 3 |
| Cancer Prevention  Study II | U.S., nationwide | 1992/2007 | General population | 160,394 | |  | 115 | |  | 31 | |
|  |  |  |  | 76,154 | 84,240 |  | 89 | 26 |  | 19 | 12 |
| Iowa Women's  Health Study | Iowa | 1986/2007 | Postmenopausal women, ages  55-69 years | 28,570 | |  | 29 | |  | 10 | |
|  |  |  |  | - | 28,570 |  | - | 29 |  | - | 10 |
| Black Women's  Health Study | multiple states | 1995/2009 | General population, self-identified  as black or African American | 57,152 | |  | 6 | |  | 1 | |
|  |  |  |  | - | 57,152 |  | - | 6 |  | - | 1 |
| Women's Health  Initiative | multiple states | 1993/variable | Postmenopausal women, ages  50-79 years | 160,988 | |  | 67 | |  | 46 | |
|  |  |  |  | - | 160,988 |  | - | 67 |  | - | 46 |
| Nurses' Health Study | multiple states | 1980/2010 | Married, female nurses, aged 30-55 | 102,459 | |  | 49 | |  | 14 | |
|  |  |  |  | - | 102,459 |  | - | 49 |  | - | 14 |
| TOTAL |  |  |  | 1,516,908 | |  | 1,423 | |  | 410 | |
|  |  |  |  | 603,416 | 913,492 |  | 1,039 | 384 |  | 223 | 187 |

**Supporting Table S2.** Assessment of cigarette smoking, Liver Cancer Pooling Project.

| Study |  | Assessment |
| --- | --- | --- |
| NIH-AARP Diet and Health Study | | **Have you ever smoked 100 or more cigarettes during your entire life?**  Yes, No  **If yes, do you currently smoke cigarettes or have you stopped?** Currently smoke, stopped within last year, stopped 1 to 4 years ago, stopped 5 to 9 years ago, stopped 10 or more years ago  **If yes, how many cigarettes per day do or did you usually smoke?**  1 to 10, 11 to 20, 21 to 30, 31 to 40, 41 to 60, 61 or more |
| Agriculture Health Study | | **During your lifetime, have you smoked at least 100 cigarettes?** No, Yes **Do you smoke cigarettes now?** No, Yes **On the average, how much do you or did you smoke each day?** 10 cigarettes or less 11–20 cigarettes 21–40 cigarettes More than 40 cigarettes **What is the total number of years you smoked cigarettes?** (Enter years) |
| United States Radiologic Technologists | | **Have you ever SMOKED cigarettes regularly for a period of one year or more?**  No, Yes  **Excluding any periods of time during which you did not smoke, how many total years have you smoked regularly?**  (Enter total years smoked)  **During the time you smoked, regularly, about how many cigarettes per day did you smoke?**  (Enter no. of cigarettes per day)  **How old were you when you started smoking regularly?** (Enter age started)   **How old were you when you stopped smoking?**  Currently smoking (Enter age stopped) |
|  | |  |
|  | |  |
|  | |  |
|  | |  |
|  | |  |
|  | |  |
|  | |  |
|  | |  |
|  | |  |
| **Study** | | **Assessment** |
| The Breast Cancer Detection Demonstration Project (cont.) | | **Have you smoked a total of 100 cigarettes or more in your lifetime?**  Yes, No  **How old were you when you first started smoking cigarettes regularly?** (Enter age first started smoking)  **Do you smoke cigarettes now?**  Yes, No  **How old were you when you last stopped smoking cigarettes?** (Enter age last stopped smoking cigarettes)  **Considering the time you may have stopped smoking and then restarted, how many total years have you or did you actually smoke cigarettes?** (Enter total years smoked cigarettes)  **During the time you have smoked regularly, how many cigarettes do or did you usually smoker per day?**  (Enter number of cigarettes per day) |
| Prostate, Lung, Colorectal and Ovarian Cancer Screening Trial | | **Have you ever smoked cigarettes regularly for six months or longer?** No, Yes  **At what age did you start smoking cigarettes regularly?**  (Enter age in years)  **Do you smoke cigarettes regularly now?** No, Yes  **At what age did you last stop smoking cigarettes regularly?** (Enter age in years)  **During periods when you smoked, how many cigarettes did or do you usually smoke per day?** 1-10, 11-20, 21-30, 31-40, 41-60, 61-80, 81 or more |
| Women’s Health Study | | **Have you smoked 100 cigarettes or more in your lifetime?** No; Yes, currently smoke; Yes, smoked in past but quit  **IF YES, BUT QUIT: When did you quit?**  Less than 1 year ago, 1 or more years ago  **How many total years have you smoked?** Less than 5 years, 5-9, 10-19, 20-29, 30-39, 40-49, 50 or more years  **At each age: what was the average number of cigarettes you smoked PER DAY during the time that you smoked?** AGE: <15, 15-19, 20-29, 30-39, 40-49, 50-59, 60-69, 70+ None, 1-4, 5-14, 15-24, 25-35, 36-44, 45+ |
|  | |  |
|  | |  |
|  | |  |
|  | |  |
| **Study** | | **Assessment** |
| Physicians’ Health Study | | **Have you ever smoked cigarettes regularly?** Never, Past only, Current  **IF CURRENT: On average how many cigarettes do you currently smoke daily?**  (Enter number of cigarettes) |
| Health Professionals Follow-up Study | | **Have you smoked 20 packs of cigarettes or more in your lifetime?** No; Yes, currently smoke; Yes, smoked in past but quit  **IF YES, BUT QUIT: When did you quit?**  <1 year, 1-2 years, 3-5 years, 6-8 years, 10+ years  **At each age: average number of cigarettes per day** AGE: <15, 15-19, 20-29, 30-39, 40-49, 50-59, 60+ None, 1-4, 5-14, 15-24, 25-35, 36-44, 45+ |
| NYU Women's Health Study | | **DID YOU EVER SMOKE CIGARETTES?** No, Yes  **IF YES: At what age did you start?**  (Enter age)  **Are you CURRENTLY smoking?**  No, Yes  **IF NO: When did you stop?** (Enter age)  **Did you ever stop TEMPORARILY and then start up again?**  No, Yes  **IF YES: For how many years altogether did you stop smoking temporarily?**  (Enter years)  **How many cigarettes do/did you usually smoke PER DAY?** (Enter cigarettes/day) |
|  | |  |
|  | |  |
|  | |  |
|  | |  |
|  | |  |
|  | |  |
|  | |  |
|  | |  |
|  | |  |
|  | |  |
|  | |  |
|  | |  |
|  | |  |
|  | |  |
|  | |  |
| **Study** | | **Assessment** |
| Cancer Prevention Study–II Nutrition Cohort | | **Have you smoked at least 100 cigarettes in your lifetime?**  No, Yes  **Do you smoke cigarettes now?** Yes, No  **IF YES:  On average, about how many cigarettes a day do you smoke?**  (Enter number of cigarettes)  **How many years have you smoked?**  (Enter years)  **How many times since you first started smoking have you quit smoking for 6 months or longer?** (Enter times)  **IF NO:  How old were you the last time you quit smoking cigarettes?** (Enter age)  **On average, how many cigarettes a day did you smoke?**  (Enter number of cigarettes)  **How many years did you smoke?** (Enter years)  **During the years that you were smoking, how many times did you quit smoking for 6 months or longer?**  (Enter number of times) |
| Iowa Women’s Health Study | | **Have you ever smoked cigarettes on a regular basis, that is, more than 100 cigarettes in your entire life?**  No, Yes  **How old were you when you first started smoking cigarettes on a regular basis?** (Enter age)  **On average, during the entire time you smoked, how many cigarettes did you smoke per day?** (Enter cigarettes/day)  **Do you smoke cigarettes now?** No, Yes  **How old were you when you stopped smoking?** (Enter age)  **On average, about how many cigarettes a day do you currently smoke?** (Enter cigarettes/day) |
|  | |  |
|  | |  |
|  | |  |
|  | |  |
| **Study** | | **Assessment** |
| Black Women’s Health Study | | **Have you ever smoked one cigarette or more every day for at least a year?**  Yes, I smoke currently; Yes, but I no longer smoke; No  **At what age did you start to smoke regularly?** 14 or less, 15, 16, 17, 18, 19, 20, 21 or more  **In the *first few years* that you smoked, how many cigarettes did you smoke each day?** Less than 5, 5-14, 15-24, 25-34, 35-44, 45 or more  **In the *last few years* that you smoked, how many cigarettes did you smoke each day?** Less than 5, 5-14, 15-24, 25-34, 35-44, 45 or more  **If you have stopped smoking, when did you stop?** Less than 1 year ago, 1 year ago, 2 years ago, 3-4 years ago, 5-9 years ago, 10 or more years ago  **How many years have you been smoking or did you smoke in the past?** Less than 10, 10-14, 15-19, 20-24, 25-29, 30 or more |
| Women’s Health Initiative | | **During your entire life, have you smoked at least 100 cigarettes?** No, Yes  **How old were you when you first started smoking cigarettes regularly?**  Less than 15, 15-19, 20-24, 25-29, 30-34, 35-39, 40-44, 45-49, 50-54, 55-59, 60 or older  **On average, how many cigarettes do you (did you) usually smoke each day?** Less than 1, 1-4, 5-14, 15-24, 25-34, 35-44, 45 or more  **How many years have you been (were you) a regular smoker?** Less than 5 years, 5-9 years, 10-19 years, 20-29 years, 30-39 years, 40-49 years, 50 or more years |
|  | |  |
|  | |  |
|  | |  |
|  | |  |
|  | |  |
|  | |  |
|  | |  |
|  | |  |
|  | |  |
|  | |  |
|  | |  |
|  | |  |
|  | |  |
|  | |  |
|  | |  |
|  | |  |
|  | |  |
|  | |  |
|  | |  |
|  | |  |
|  | |  |
| **Study** | | **Assessment** |
| Nurses' Health Study | | **Do you smoke cigarettes CURRENTLY?** Yes, No  **IF YES: On average, how many cigarettes do you smoke currently each day?**  (Enter cigarettes per day) **How old were you when you first started to smoke regularly?** (Enter age)  **Since you first started to smoke regularly, have you ever given up smoking cigarettes for 6 months or more?** Yes, No  **IF NO:  Have you ever smoked cigarettes regularly in the past?** Yes, No  **On average, how many cigarettes per day did you smoke when you last smoked regularly?** (Enter cigarettes per day)  **How old were you when you last smoked regularly?** (Enter age)  **How old were you when you first started to smoke regularly?** (Enter age) **From when you first started to smoke regularly until you last smoked regularly, did you ever give up smoking for 6 months or more?** Yes, No |

**Supporting Table S3.** Assessment of alcohol consumption, Liver Cancer Pooling Project.

| **Study** |  | **Assessment** |
| --- | --- | --- |
| NIH-AARP Diet and Health Study |  | **Over the last 12 months, how often did you eat the following foods? (Ignore any recent changes.)** **HOW OFTEN:** Never, 1 time per month or less, 2-3 times per month, 1-2 times per week, 3-4 times per week, 5-6 times per week, 1 time per day, 2-3 times per day, 4-5 times per day, 6+ times per day **Beer (IN SUMMER)** HOW MUCH: Less than a 12-ounce can, 1 to 2 12-ounce cans, more than 2 12-ounce cans **Beer (REST OF THE YEAR)** HOW MUCH: Less than a 12-ounce can, 1 to 2 12-ounce cans, more than 2 12-ounce cans **Wine or wine coolers** HOW MUCH: Less than 4 ounces, 4 to 8 ounces, more than 8 ounces **Liquor of mixed drinks** HOW MUCH: Less than 1 shot of liquor, 1 to 2 shots of liquor, more than 2 shots of liquor |
| Agriculture Health Study |  | **For these questions, a drink is defined as one beer, a glass of wine, or a shot of hard liquor.  During the past 12 months, how often did you usually drink any kind of alcoholic beverage?**  Never, less than one time a month, 1-3 times a month, 1 time a week, 2-4 times a week, almost every day, every day  **During the past 12 months, about how many drinks would you have on a day when you drank?**  Didn't drink last year, 1 or 2 drinks, 3 or 4 drinks, 5-8 drinks, 9 or more drinks |
| United States Radiologic  Technologists |  | **During the past year, how often did you drink the following BEVERAGES? Beer** (12 oz.) **Wine** (4 oz.) **Liquor** (1 shot) **AVERAGE NUMBER OF SERVINGS CONSUMED DURING PAST YEAR:** Never, <1/month, 1/month, 2-3/month, 1/week, 2/week, 3-4/week, 5-6/week, 1/day, 2/day, 3/day, 4/day, 5+/day |
| The Breast Cancer Detection  Demonstration Project |  | **Have you ever drunk alcoholic beverages, such as beer, wine, or liquor regularly, that is, at least once a month?**  Yes, No  **Have you ever drunk beer regularly, that is, at least once a month?**  Yes, No  **Between the ages of 30 and 50, what was or has been the usual number of beers you had per week?** Number per week: (fill in) Less than one per week Never drank beer between ages 30 and 50  **Have you ever drunk wine regularly, that is, at least once a month?**  Yes, No  **Between the ages of 30 and 50, how many glasses of wine do or did you usually drink per week?** Number per week: (fill in) Less than one per week Never drank wine between ages 30 and 50  **Have you ever drunk liquor, or drinks containing liquor, regularly, that is, at least once a month?**  Yes, No  **Between the ages of 30 and 50, what was or is the unusual number of drinks containing liquor you had per week?** Number per week: (fill in) Less than one per week Never drank liquor between ages 30 and 50 |
|  |  |  |
| **Study** |  | **Assessment** |
| Prostate, Lung, Colorectal and  Ovarian Cancer Screening Trial |  | **Over the past 12 months, did you drink beer?** No, Yes  **If yes, how often did you drink beer IN THE SUMMER?** NEVER, 1 time per month or less, 2-3 times per month, 1-2 times per week, 3-4 times per week, 5-6 times per week, 1 time per day, 2-3 times per day, 4-5 times per day, 6 or more times per day  **If yes, how often did you drink beer DURING THE REST OF THE YEAR?** NEVER, 1 time per month or less, 2-3 times per month, 1-2 times per week, 3-4 times per week, 5-6 times per week, 1 time per day, 2-3 times per day, 4-5 times per day, 6 or more times per day  **If yes, each time you drank beer, how much did you usually drink?** Less than a 12-ounce can or bottle, 1 to 3 12-ounce cans or bottles, more than 3 12-ounce cans or bottles  **Over the past 12 months, how often did you drink wine or wine coolers?**  NEVER, 1 time per month or less, 2-3 times per month, 1-2 times per week, 3-4 times per week, 5-6 times per week, 1 time per day, 2-3 times per day, 4-5 times per day, 6 or more times per day  **If yes, each time you drank wine or wine coolers, how much did you usually drink?** Less than a 5 ounces or less than 1 glass, 5 to 12 ounces or 1 to 2 glasses, more than 12 ounces or more than 2 glasses  **Over the past 12 months, how often did you drink liquor or mixed drinks?** NEVER, 1 time per month or less, 2-3 times per month, 1-2 times per week, 3-4 times per week, 5-6 times per week, 1 time per day, 2-3 times per day, 4-5 times per day, 6 or more times per day  **If yes, each time you drank liquor or mixed drinks, how much did you usually drink?** Less than 1 shot of liquor, 1 to 3 shots of liquor, more than 3 shots of liquor |
| Women’s Health Study |  | **Please record your average consumption of the following beverages over the LAST YEAR:** **Beer** (1 glass, bottle, can) **Red wine** (include sherry, port) (4 oz. glass) **White wine** (4 oz. glass) **Liquor** (e.g., vodka, rum, gin, liqueur, brandy) (one drink or shot)  Never or less than one/month, 1-3 per month, 1 per week, 2-4 per week, 5-6 per week, 1 per day, 2-3 per day, 4-5 per day, 6+ per day |
| Physicians’ Health Study |  | **How often do you consume alcoholic beverages (beer, wine, liquor)?** 2+/day, daily, 5-6/wk, 2-4/wk, 1/wk, 1-3/mo, rarely/never |
| Health Professionals Follow-up Study |  | **Please fill in your average use, during the past year, of each specified food.** **Beer** (1 glass, bottle, can) **Red wine** (4 oz glass) **White wine** (4 oz glass) **Liquor, e.g. whiskey, gin, etc.** (1 drink or shot)  Never or less than one/month, 1-3 per month, 1 per week, 2-4 per week, 5-6 per week, 1 per day, 2-3 per day, 4-5 per day, 6+ per day |
|  |  |  |
|  |  |  |
|  |  |  |
| **Study** |  | **Assessment** |
| NYU Women's Health Study |  | **ABOUT TEN YEARS AGO, approximately how many cans or bottles of beer did you usually drink in a week?** (# of cans/bottles per week)  **ABOUT TEN YEARS AGO, approximately how many 4-ounce glasses of wine did you usually drink in a week?** (# of 4-ounce glasses per week)  **ABOUT TEN YEARS AGO, approximately how many 1-ounce glasses (shots) of liquor, either straight or in a mixed drink, did you usually drink in a week?** (# of 1-ounce glasses per week) |
| Cancer Prevention Study–II  Nutrition Cohort |  | **Complete the following list to reflect your USUAL eating habits over the past year (at home, in restaurants, and other places): TYPE OF FOOD:** **Beer** (MEDIUM SERVING SIZE: 12 ounces) **Wine or wine coolers** (MEDIUM SERVING SIZE: 1 glass) **Liquor** (MEDIUM SERVING SIZE: 1 shot)  **YOUR SERVING SIZE:** S, M, L **AVERAGE USE LAST YEAR:** Never or less than once per month, 1-3 per month, 1 per week, 2-4 per week, 5-6 per week, 1 per day, 2-3 per day, 4-5 per day, 6+ per day |
| Iowa Women’s Health Study |  | **Please fill in your average use during the past year, of each specified food.**  **Beer** (1 glass, bottle, can) **Red Wine** (4 oz. glass) **White Wine** (4 oz. glass) **Liquor, e.g. whiskey, gin, etc.** (1 drink or shot)  Never or less than once per month, 1-3 per mo., 1 per week, 2-4 per week, 5-6 per week, 1 per day, 2-3 per day, 4-5 per day, 6+ per day |
| Black Women’s Health Study |  | **Did you ever drink alcoholic beverages (beer, wine, wine cooler, or liquor) at least once a week for at least a year?** Yes, I drink currently; Yes, but I no longer drinker; No  **In the *past year*, how many drinks of beer, wine (or wine cooler) and liquor did you drink each *week*? Beer** (12 oz.) **Wine** (4 oz.) **Liquor** (1 shot) **Number of drinks per week:** Less than 1, 1-3, 4-6, 7-13, 14-20, 21 or more |
| Women’s Health Initiative |  | **During the last three (3) months, how often did you eat these foods?  Beer** (medium serving size = 12 ounce can or bottle) **Wine** (medium serving size = 1 medium glass [6 ounces]) **Liquor** (medium serving size = 1 shot [1 1/2 ounces]) Never or less than once per month, 1-3 per mo., 1 per week, 2-4 per week, 5-6 per week, 1 per day, 2-3 per day, 4-5 per day, 6+ per day Your serving size: S M L |
|  |  |  |
|  |  |  |
|  |  |  |
|  |  |  |
| **Study** |  | **Assessment** |
| Nurses' Health Study |  | **Please fill in your average use during the past year, of each specified food.**  **Beer** (1 glass, bottle, can) **Red Wine** (4 oz glass) **White Wine** (4 oz glass) **Liquor, e.g. whiskey, gin, etc.** (1 drink or shot)  Never or less than once per month, 1-3 per mo., 1 per week, 2-4 per week, 5-6 per week, 1 per day, 2-3 per day, 4-5 per day, 6+ per day |

**Supporting Table S4.** Adjusted* Hazard Ratios (HR) and 95% Confidence Intervals (CI) for Associations Between Any Alcohol Intake, with Light Drinkers as the Referent, and Hepatocellular Carcinoma and Intrahepatic Cholangiocarcinoma, Liver Cancer Pooling Project.

**Supporting Table S5.** Adjusted* Hazard Ratios (HR) and 95% Confidence Intervals (CI) for Interaction between Alcohol Consumption and Other Risk Factors and Hepatocellular Carcinoma Risk, Liver Cancer Pooling Project.

**Supporting Table S6.** Adjusted* Hazard Ratios (HR) and 95% Confidence Intervals (CI) for Interaction between Cigarette Smoking and Other Risk Factors and Hepatocellular Carcinoma Risk, Liver Cancer Pooling Project.

**Supporting Table S7.** Adjusted* Hazard Ratios (HR) and 95% Confidence Intervals (CI) for Interaction between Alcohol Consumption and Other Risk Factors and Intrahepatic Cholangiocarcinoma Risk, Liver Cancer Pooling Project.

**Supporting Table S8.** Adjusted* Hazard Ratios (HR) and 95% Confidence Intervals (CI) for Interaction between Cigarette Smoking and Other Risk Factors and Intrahepatic Cholangiocarcinoma Risk, Liver Cancer Pooling Project.

**Supporting Table S9.** Adjusted* Hazard Ratios (HR) and 95% Confidence Intervals (CI) for Associations Between Cigarette Smoking and Hepatocellular Carcinoma and Intrahepatic Cholangiocarcinoma Incidence, 5-year Lag-Analysis, Liver Cancer Pooling Project.

**Supporting Table S10.** Adjusted* Hazard Ratios (HR) and 95% Confidence Intervals (CI) for Associations Between Alcohol Intake and Hepatocellular Carcinoma and Intrahepatic Cholangiocarcinoma Incidence, 5-year Lag-Analysis, Liver Cancer Pooling Project.

**Supporting Table S11.** Adjusted* Hazard Ratios (HR) and 95% Confidence Intervals (CI) for Associations Between Smoking and Alcohol Intake and Hepatocellular Carcinoma and Intrahepatic Cholangiocarcinoma Incidence by Follow-Up Time, Liver Cancer Pooling Project.

**Supporting Table S12.** Adjusted* Hazard Ratios (HR) and 95% Confidence Intervals (CI) for Associations Between Cigarette Smoking and Confirmed or Suspected Hepatocellular Carcinoma, Liver Cancer Pooling Project.

**Supporting Table S13.** Adjusted* Hazard Ratios (HR) and 95% Confidence Intervals (CI) for Associations Between Alcohol Intake and Confirmed or Suspected Hepatocellular Carcinoma, Liver Cancer Pooling Project.
